# Induction of Innate Immune Memory in LPS-Primed Microglial Cells by Water-Soluble Chitosan

**DOI:** 10.1155/bmri/8027006

**Published:** 2024-11-30

**Authors:** Vo Thuy Anh Thu, Thi Xoan Hoang, Jae Kweon Park, Jae Young Kim

**Affiliations:** Department of Life Science, Gachon University, Seongnam, Gyeonggi-do 13120, Republic of Korea

**Keywords:** microglia, trained immunity, water-soluble chitosan

## Abstract

Innate immune memory or trained immunity refers to a long-lasting response of the innate immune cells against repeated exposure to the homogenous or heterogenous infectious agent. The trained immunity is induced through epigenetic modification and is characterized by the change of both intracellular immunological signaling and cellular metabolism. Recently, different groups have tried to establish protocols to generate trained innate immune cells. However, the molecular basis of innate memory induction remains poorly understood. Here, we evaluated the impact of water-soluble chitosan on the innate immune memory induction in microglial cells primed with LPS. The trained-immune response was accessed by measuring proinflammatory markers, metabolic change, and epigenetic modification. We showed that the stimulation/restimulation with LPS only caused a robust reduction of iNOS, and proinflammatory cytokines, indicating induced immune tolerance. In contrast, the treatment of chitosan induces long-lasting memory microglial cells accompanied by a high level of iNOS, increased lactate production, induced epigenetic modification, and the upregulation of proinflammatory cytokines upon further exposure to the same stimulus. These findings suggest that chitosan induces microglial-trained immunity by targeting distinct epigenetic and metabolic pathways; therefore, chitosan treatment may provide a novel approach for targeting innate immunity towards a memory-like response in an in vitro model.

## 1. Introduction

The objective of this study is to investigate the impact of chitosan on the generation of trained immunity in human microglial cells. The emergence of trained immunity, a form of innate immune memory, has revolutionized our understanding of how innate immune cells enhance their response upon subsequent pathogen encounters. This phenomenon involves heightened cytokine production and profound metabolic and epigenetic reprogramming following an initial inflammatory stimulus, enabling a robust response to future infections [1–3]. Distinct from the adaptive immune system's reliance on gene recombination, trained immunity in mammals is characterized by epigenetic and transcriptional changes, supported by metabolic shifts that provide essential substrates for chromatin-modifying enzymes, without altering the genomic sequence [4–6].

Microglia, the brain's resident immune cells, play a crucial role in regulating immune responses in the central nervous system. Due to the brain's limited regenerative capacity, microglia are tightly controlled and can undergo long-lasting epigenetic changes in response to inflammatory stimuli. This process, known as microglia priming, leads to heightened reactivity to future challenges, resembling trained immunity observed in other immune cells. Understanding trained immunity in microglia is key to developing therapies for neurodegenerative diseases, where chronic inflammation is a major [7–9].

Lipopolysaccharide (LPS), a well-characterized pathogen–associated molecular pattern (PAMP), is widely used in research to mimic bacterial infections and induce inflammatory responses in immune cells, including microglia. LPS is an effective priming agent because it reliably activates the toll-like receptor 4 (TLR-4) pathway, which is essential for both immediate immune response and the establishment of long-term immune memory.

Chitosan, a cationic polysaccharide known for its immunomodulatory properties, has shown potential in modulating innate immune responses, including the activation of macrophages and stimulation of cytokine secretion [10, 11]. Its engagement with immune pathways, such as the cGAS-STING pathway in dendritic cells, underscores its potential in influencing trained immunity [12]. Additionally, chitosan has been used as a biomaterial nanoparticle fabrication to counteract LPS-induced damage in rat astrocytes, and it has been shown to enhance trained immunity in other immune cell types [13]. However, the mechanisms by which chitosan induces trained immunity in microglial cells remain unclear, and its potential therapeutic applications warrant further investigation.

In this study, we aimed to investigate the impact of chitosan on the trained immunity of human microglial cells, focusing on its potential to modulate epigenetic and metabolic pathways involved in immune memory formation. We combined LPS priming with chitosan treatment to examine how this natural compound enhances immune responses in microglia. Our findings demonstrate that chitosan not only boosts inducible nitric oxide synthase (iNOS) expression, a marker of enhanced immune protection, but also regulates key epigenetic markers, including H3K4 and H3K9 methyltransferases, while increasing the levels of lactate dehydrogenase A (LDHA), which is essential for lactate production and macrophage activation. These results suggest that chitosan augments microglial-trained immunity by targeting specific epigenetic and metabolic pathways, highlighting its potential therapeutic applications in neuroimmunology and beyond [2, 14].

## 2. Methods

### 2.1. Preparation of Water-Soluble Chitosan (WSC) Oligosaccharide

WSC oligosaccharides were synthesized through enzymatic hydrolysis of high molecular weight water-soluble chitosan (HMW-WSC) by slightly modifying the previous method [15]. Briefly, the HMW-WSC, provided by Sok Cho Ltd. (Sok Cho, Korea), had a deacetylation degree estimated at approximately 82% and a molecular weight of 600 kDa as determined by the supplier. To prepare the WSC, a 2% chitosan solution was created by dissolving 2 g of HMW-WSC in 100 mL of phosphate-buffered saline (PBS). Chitosanase, acquired from Sigma-Aldrich (St. Louis, MO, USA) as a purified enzyme from *Streptomyces* sp., was added to the solution at a concentration of one unit per reaction mixture. The mixture was then incubated at 37°C for 24 h to facilitate hydrolysis. Following incubation, the reaction was halted by boiling the mixture for 10 min at 100°C, effectively deactivating the enzyme. The mixture was subsequently centrifuged at 13,000 rpm for 5 min to separate insoluble residues. The clear supernatant containing hydrolyzed WSC was collected, filtered through a 0.22-*μ*m filter, and utilized as the crude product for subsequent experiments. The molecular size of WSC obtained by hydrolysis was measured using matrix-assisted laser desorption/ionization time-of-flight (MALDI-TOF) mass spectrometry, as described in the previous study [16].

### 2.2. Chemicals and Antibodies

LPS (Cat. L2630) was obtained from Sigma-Aldrich (St. Louis, MO, USA). The iNOS antibody (Cat. sc-7271) was purchased from Santa Cruz Biotechnology (Dallas, TX, USA), and the Iba1 antibody (Cat. ab178846) was produced from Abcam (Cambridge, UK). Lactate assay kit (Cat. K607-100) was purchased from Biovision (CA, USA).

### 2.3. Cell Culture

An established human microglial cell line [17], HMO6 (accession number CVCL_5G94) was cultured in Dulbecco's Modified Eagle's Medium (DMEM) supplemented with 10% heat-inactivated fetal bovine serum (FBS) and 1% antibiotic-antimycotic solution (Invitrogen Corp., Carlsbad, CA). Cells were incubated at 37°C in a humidified atmosphere containing 5% CO_2_.

### 2.4. Immune Training Protocol

**Figure 1:**
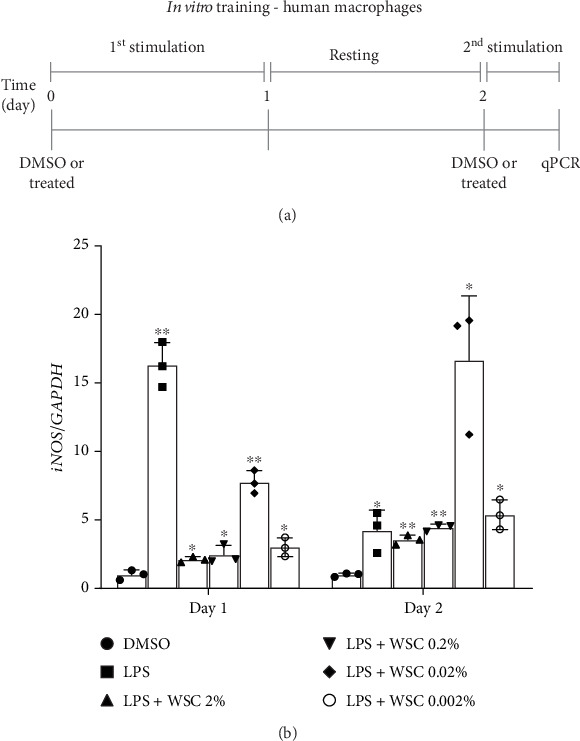
Enhancement of microglial reactivity by WSC upon secondary LPS exposure. (a) Experimental timeline: depicts the sequence of initial exposure, rest period, and restimulation to evaluate the effects of WSC on microglial activation following a second LPS challenge. (b) Gene expression analysis: quantitative real-time PCR results showing mRNA levels after the first (24 h postinitial exposure) and second stimulations (6 h post-re-exposure). Results were normalized against a DMSO control to assess changes in gene expression indicative of microglial activation or priming by WSC. ⁣^∗^*p* < 0.05 and ⁣^∗∗^*p* < 0.005 versus DMSO.

For a short-term response protocol, microglial cells were exposed to LPS (100 ng/mL) alone or in combination with varying concentrations of WSC for 24 h. Following this initial treatment, the cells were washed and incubated in fresh medium without any treatment for 24 h, simulating a rest period. Subsequently, the cells were re-exposed to the same treatments for an additional 6 h to measure mRNA expression or 12 h to measure protein expression (Figure 1(a)).

**Figure 2:**
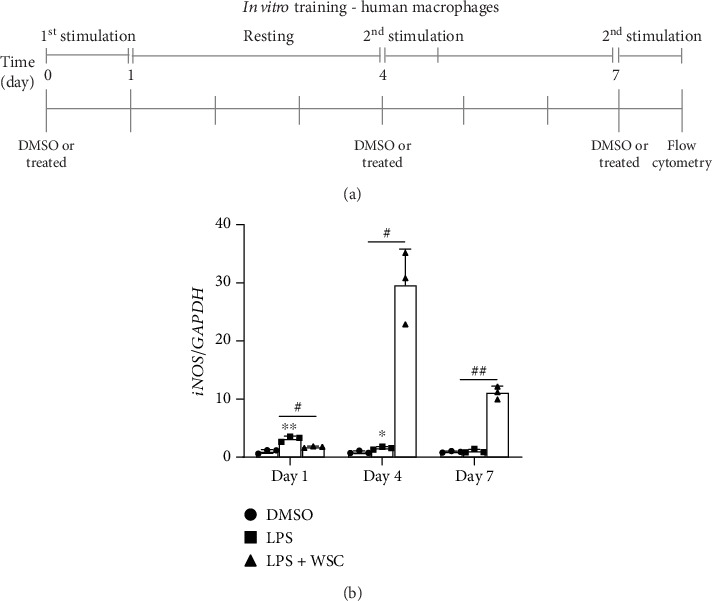
Long-term modulation of microglial reactivation by WSC post-LPS exposure. (a) Experimental design timeline: outlines the sequence of LPS and/or WSC treatment, followed by washout periods and subsequent restimulation to investigate the lasting impact of WSC on microglial activation. (b) Gene expression evaluation: quantitative real-time PCR of iNOS mRNA levels after initial treatment and 6 h post-restimulation. The comparative analysis against a DMSO baseline control highlights shifts in gene expression that indicate alterations in microglial activation and potential priming effects of WSC. ⁣^∗^*p* < 0.05 and ⁣^∗∗^*p* < 0.005 versus DMSO; #*p* < 0.05 and ##*p* < 0.005.

To evaluate long-term trained immune response, microglial cells were treated with LPS (100 ng/mL) either alone or in combination with 0.02% WSC for 24 h. After this, cells were subjected to washout periods of either 3 or 6 days, during which they were maintained in a fresh medium without any treatments to assess the long-term impact of WSC. On Day 4 or Day 7, cells were treated with the same reagents for an additional 6 h to measure mRNA expression or 12 h to measure protein expression (Figure 2(a)).

### 2.5. RNA Preparation and Real-Time Quantitative PCR

Total RNA was extracted using the easy-BLUE Total RNA Extraction Kit (iNtRON Biotechnology, Inc., Seongnam, Korea) following the manufacturer's protocol. RNA concentration was determined using a MaestroNano MicroVolume Spectrophotometer (Maestrogen, Las Vegas, NV, USA). For cDNA synthesis, 2 *μ*g of RNA was reverse-transcribed using Hyperscript 2X RT Master mix (Geneall Biotechnology, Seoul, Korea). Quantitative real-time PCR was conducted on a Rotor-Gene system (Qiagen, Hilden, Germany) utilizing the QuantiSpeed SYBR NO-ROX Kit (PhileKorea, Korea), with primers listed for each gene of interest. Following primer sets were used: GAPDH: 5⁣′-ACA GCC TCA AGA TCA TCA GCA AT-3⁣′, 5⁣′-AGG AAA TGA GCT TGA CAA AGT GG-3⁣′; iNOS: 5⁣′- ACA GCA CAT TCA GAT CCC A-3⁣′, 5⁣′-AAC ACG TTC TTG GCA TGC AT-3⁣′; SETD1B: 5⁣′- GGG TTA ACG GCA TGG AGA AC-3⁣′, 5⁣′- GAG CCG GGT CAA TCA TCA AC-3⁣′; SUV39H1: 5⁣′- CAA GCT TGG CCA ACT ACC TG-3⁣′, 5⁣′- TCT CTA CAG TGA TGC GTC CC-3⁣′; LDHA: 5⁣′- GAT TCC GGA TCT CAT TGC CAC-3⁣′, 5⁣′- CAG CTG ATC CTT TAG AGT TGC C-3⁣′; IL-1*β*: 5⁣′-GGG ATA ACG AGG CTT ATG TGC-3⁣′, 5⁣′-AGG TGG AGA GCT TTC AGT TCA-3⁣′; TNF-*α*: 5⁣′-CAG AGG GCC TGT ACC TCA TC-3⁣′, 5⁣′-GGA AGA CCC CTC CCA GAT AG-3⁣′.

### 2.6. Flow Cytometry

Posttreatment, cells were collected, washed with DPBS, and stained with primary antibodies followed by PE-conjugated secondary antibodies at 4°C for extracellular protein analysis. For intracellular staining, cells were fixed with 4% formaldehyde and permeabilized with 1% Triton X-100 before antibody incubation. Cells were then resuspended in 0.4 mL PBS for analysis using a Cytomics FC500 MLP flow cytometer (Beckman Coulter Inc., Fullerton, CA, USA).

### 2.7. Immunofluorescence

Treated HMO6 cells were fixed with 4% formaldehyde, permeabilized with 0.1% Triton X-100, and blocked with 5% BSA in PBS. Cells were incubated with Iba-1 primary antibody, followed by a FITC-conjugated secondary antibody. Nuclei were stained with Hoechst 33342, and images were captured using a fluorescent microscope (Olympus, CKX53, Tokyo, Japan).

### 2.8. Lactate Measurement

Lactate levels were determined using a lactate fluorometric assay kit according to the manufacturer's guidelines. Briefly, supernatants from treated cells were collected and mixed with the reaction mix in assay wells. After 30 min of incubation at room temperature, absorbance was read at 570 nm.

### 2.9. Statistical Analysis

All experiments were performed at least three times. Data are presented as mean ± SD. Statistical significance was determined using one-way ANOVA followed by Tukey's post hoc test, performed with SPSS 12.0 for Windows. A *p* value of < 0.05 was considered statistically significant.

## 3. Results

### 3.1. Chitosan Enhances Trained Immunity in Microglial Cells

**Figure 3:**
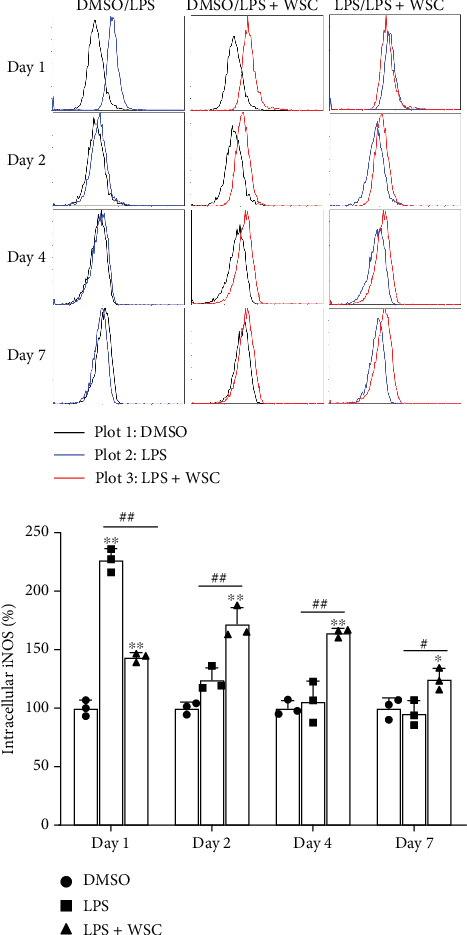
WSC amplifies microglial iNOS response to secondary LPS stimulation. Microglial cells were initially treated with LPS (100 ng/mL) alone or with 0.02% WSC for 24 h, followed by washout periods of 1, 3, or 6 days. iNOS expression was measured using flow cytometry after both initial treatment and 12 h post-restimulation, assessing WSC's impact on microglial activation. ⁣^∗^*p* < 0.05 and ⁣^∗∗^*p* < 0.01 versus DMSO; #*p* < 0.05 and ##*p* < 0.005.

Microglial cells adapt their phenotype in response to initial stimuli, developing either a trained or tolerant phenotype. This study explores how chitosan influences the development of immune memory in microglia primed with LPS. We observed a variable response to different concentrations of WSC combined with a fixed LPS dose, particularly noting an upregulation in the expression of iNOS, a proinflammatory marker, with 0.02% WSC on Day 2, indicating a shift towards a trained phenotype (Figure 1). This contrasts with the reduction in iNOS expression following re-exposure to LPS alone, which aligns with the establishment of immune tolerance [18, 19]. Further analysis showed that prolonged exposure to LPS, with or without 0.02% WSC, led to different iNOS expression outcomes. Only the combination of LPS and WSC significantly elevated iNOS expression levels after a 3-day and 6-day interval postinitial stimulation, suggesting sustained trained immunity (Figure 2). Protein level measurements corroborated the gene expression findings, with WSC-treated cells showing significantly higher iNOS levels after long-term LPS exposure, underscoring WSC's potential to strengthen microglial responses to subsequent LPS challenges (Figure 3).

### 3.2. Chitosan Modulates Epigenetic and Metabolic Pathways in LPS-Primed Microglia

**Figure 4:**
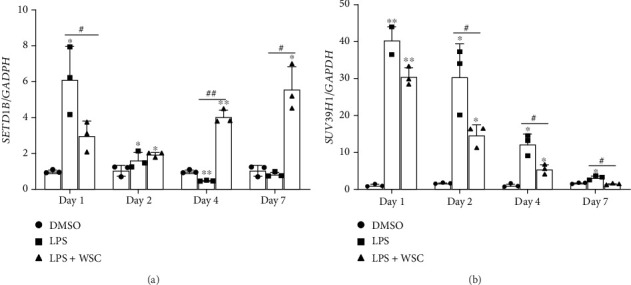
WSC modulates LPS-induced epigenetic factor expression in human microglia. Microglial cells were treated with LPS (100 ng/mL) alone or with 0.02% WSC for 24 h, followed by washout periods of 1, 3, or 6. mRNA expression of histone methyltransferases (a) *SETD1B* and (b) *SUV39H1* was measured by qPCR after initial stimulation and 6 h post-restimulation. ⁣^∗^*p* < 0.05 and ⁣^∗∗^*p* < 0.005 versus DMSO; #*p* < 0.05 and #*p* < 0.001.

**Figure 5:**
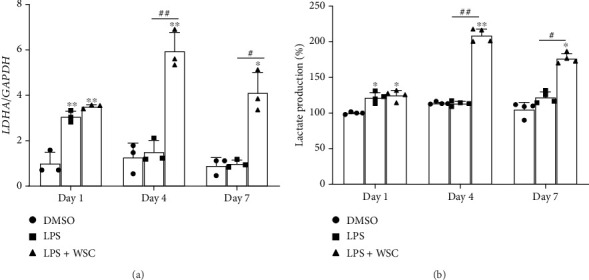
Influence of WSC on LPS-induced lactate production in human microglia. Microglial cells were exposed to LPS (100 ng/mL) alone or with 0.02% WSC for 24 h, followed by 3 or 6 washouts. (a) *LDHA* mRNA expression was measured after the first 24-h stimulation 6 h restimulation. (b) The concentration of lactate in the culture supernatant was determined using a lactate fluorometric assay kit after both the first and second 24-h stimulation periods. ⁣^∗^*p* < 0.05 and ⁣^∗∗^*p* < 0.005 versus DMSO; #*p* < 0.05 and ##*p* < 0.005.

Our investigation extended to the epigenetic mechanisms underpinning the trained immune response, focusing on the expression of genes encoding H3K4me3 methyltransferase (SETD1B) and H3K9 methyltransferases (SUV39H1). WSC treatment notably upregulated *SETD1B* expression, diverging from the suppressive effect of LPS alone, highlighting SETD1B's role in the chitosan–induced trained immunity (Figure 4(a)). Conversely, WSC treatment resulted in a significant downregulation of *SUV39H1* (Figure 4(b)). Metabolic reprogramming towards increased lactate production, a key feature of trained immunity, was also observed. WSC treatment upregulated *LDHA* gene expression, responsible for converting pyruvate to lactate, indicating enhanced glycolytic activity in WSC–enhanced trained microglia (Figure 5(a)). Lactate production measurements further confirmed the metabolic shift induced by WSC, emphasizing its role in sustaining trained immunity (Figure 5(b)).

### 3.3. Chitosan Promotes Activation and Proinflammatory Memory in Microglial Cells

**Figure 6:**
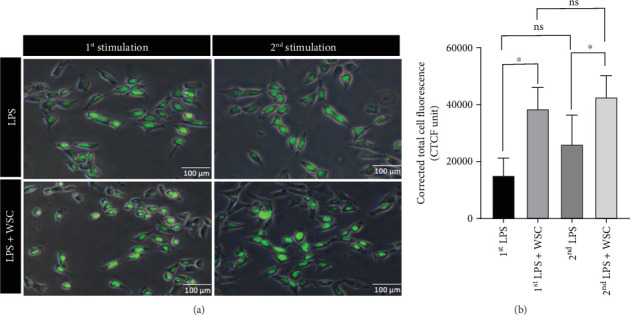
WSC augments microglial activation in response to LPS. Microglial cells were subjected to LPS (100 ng/mL) alone or with 0.02% WSC for a 6-h, followed by a 24-h washout and restimulation for 6 h. After restimulation, cells were fixed with formaldehyde, permeabilized with Triton X-100, and incubated with an anti-Iba1 antibody. (a) Fluorescence signals were captured using a fluorescent microscope at 200x magnification, with scale bars representing 100 *μ*m. (b) The corrected total cell fluorescence (CTCF) was quantitatively assessed using ImageJ software, providing a measure of Iba1 expression levels indicative of microglial activation. ⁣^∗^*p* < 0.01; ns denotes nonsignificant differences.

**Figure 7:**
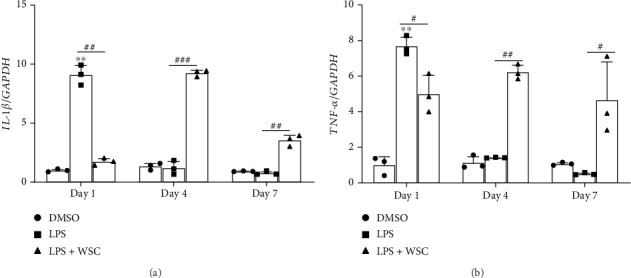
WSC amplifies proinflammatory cytokine expression in microglia following LPS challenge. Microglial cells were treated with LPS (100 ng/mL) alone or with 0.02% WSC for 24 h, followed by a washout and restimulation with the same treatments. (a) mRNA level of *IL-1β* (a) and *TNF*-*α* (b) were measured via qPCR after the first 24-h stimulation and 6 h post-restimulation. ⁣^∗^*p* < 0.05 and ⁣^∗∗^*p* < 0.005 versus DMSO; #*p* < 0.05, ##*p* < 0.005, and ###*p* < 0.001.

Assessment of Iba-1, a marker for microglial activation, revealed significant upregulation in WSC-treated, LPS-primed microglia, indicating enhanced inflammatory response capability (Figure 6). Additionally, we observed a significant upregulation in the expression of proinflammatory cytokines *IL-1β* and *TNF-α* following WSC treatment, which was not seen with LPS restimulation alone. This suggests that WSC establishes a more robust, memory-like inflammatory response in microglia, especially after long-term treatment (Figure 7).

## 4. Discussion

**Figure 8:**
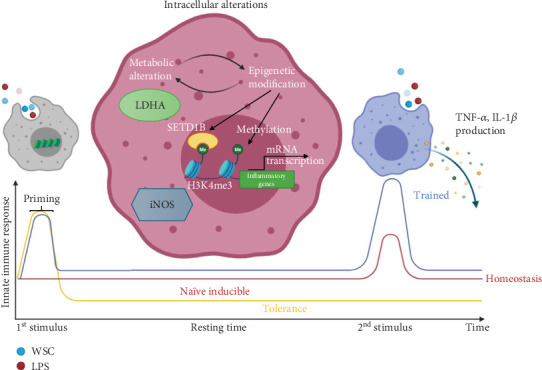
Conceptual model of enhanced microglial immune response to LPS via trained immunity induced by WSC. The model depicts three distinct phenotypic responses: endotoxin tolerance (yellow line): represents a reduced inflammatory response to repeated LPS exposure, marked by decreased cytokine production, which is commonly referred to as endotoxin tolerance. naïve inducible response (red line): illustrates the typical acute inflammatory response of naïve microglial cells to initial LPS exposure, marked by significant cytokine secretion and activation. Trained immunity (blue line): demonstrates the enhanced microglial response to a second LPS challenge in the presence of WSC, characterized by increased cytokine production, epigenetic modifications, and metabolic reprogramming.

This investigation represents the first in vitro study into WSC's role in eliciting a trained immunity phenotype in microglial cells. Our findings reveal that WSC treatment fosters immune memory formation, highlighted by the upregulation of LDHA and methyltransferase genes, which are central to the mechanisms of trained immunity (Figure 8). This response is notably amplified following LPS stimulation, underscoring the robustness of the trained immunity concept and its operational dynamics within microglia [9, 20]. Consistent with prior studies on macrophages, where chitosan induced varying responses ranging from anti-inflammatory to proinflammatory depending on the cell state and dose [21], our results support a proinflammatory phenotype in microglia.

The biological activity of chitosan depends on its molecular weight and degree of deacetylation. As an example, the immunopotentiation of chitosan oligosaccharides (COSs) is described using a mouse model. COS, derived from the enzymatic treatment of high-molecular-weight chitosan (~2000 kDa, deacetylation degree of about 98%), is a small molecule of less than 1 kDa composed of glucosamine (GlcN)_n_, *n* = 3 − 5 [22]. The production of COS below 1 kDa is relatively straightforward but rather requires the establishment of efficient production methods for single molecules with specific molecular weights. So far, we have developed or improved physical decomposition methods using ultrasound, chemical hydrolysis methods, and enzymatic treatment methods to decompose chitosan. However, it was not significantly different from what was described in various existing reports. However, as an innovative method, the main product (GlcN)_n_, *n* = 2 ~ 6, was obtained using the enzyme immobilization method [23]. Through the establishment and development of these various methods, we aimed to achieve greater progress in the study of COS production, biological applications, and properties of different molecular sizes. To prepare a solution for enzymatic treatment of high molecular weight chitosan, acetic acid, or organic acid within 2% is mainly used (pH ~4). However, for in vitro or in vivo applications, it should not have a significant impact on the growth conditions of specific cells. To solve this problem, the WSC used in this study was synthesized through an enzyme treatment method and dissolved in nonbuffered PBS to obtain WSC of various molecular sizes. As a result, the molecular weight range of WSC used in this study was confirmed to be (GlcN)_n_, *n* = 5–15, using MALDI-TOF mass spectrometry (data not shown) [24].

The upregulation of epigenetic marks, such as H3K4me3, in LPS-primed microglia treated with WSC points to a crucial role for epigenetic reprogramming in establishing immune memory. Our data show that WSC promotes a proinflammatory state by enhancing lactate production and histone methyltransferase activity, consistent with the trained immunity concept [13, 19, 25, 26]. These findings invite further exploration into how WSC-mediated metabolic reprogramming and epigenetic modifications coalesce to sustain trained immunity.

The upregulation of Iba-1, recognized as a marker for microglial activation, further validates the induction of a trained immune response by WSC treatment. The increased Iba-1 expression, indicative of an enhanced microglial activation state, aligns with our understanding of trained immunity as an accumulative process, where prior activations prime cells for heightened responses to subsequent stimuli [27–30].

Our study also observed significant upregulation of iNOS in WSC-treated microglia, indicative of a proinflammatory, trained immune phenotype. Previous research has demonstrated chitosan's ability to activate macrophages and stimulate cytokine secretion, including IL-1*β* and TNF-*α*, which play a key role in the inflammatory response. The upregulation of iNOS following WSC treatment mirrors chitosan's known activation of immune pathways such as NF-*κ*B and MAPK, which are involved in iNOS expression.

Interestingly, while LPS restimulation alone led to reduced iNOS levels, indicative of immune tolerance, the combination of WSC and LPS maintained elevated iNOS expression even after prolonged exposure. This suggests that WSC not only enhances the initial microglial activation but also sustains it over time, a departure from other studies where chitosan was primarily associated with short-term immune activation. This sustained proinflammatory response reflects WSC's potential role in modulating both metabolic and epigenetic reprogramming essential for trained immunity.

The broader implications of these findings extend beyond neuroinflammatory diseases such as Alzheimer's, Parkinson's, and multiple sclerosis, where dysregulated immune responses contribute to disease progression. WSC's ability to modulate microglial activity through trained immunity suggests its therapeutic potential in promoting more controlled immune responses or enhancing the clearance of neurotoxic substances. Furthermore, WSC-induced immune memory could have applications in transplantation, cancer immunotherapy, chronic infections, and autoimmune diseases, where fine-tuned control over immune responses is crucial.

Despite the novel insights gained from our study, we acknowledge several limitations. A significant one is the use of an immortalized microglial cell line instead of primary cells or in vivo models. While immortalized cell lines are advantageous due to their reproductivity and ease of manipulation, they may not fully replicate the complexity of microglial behavior in the brain, where factors such as the brain's microenvironment and cell-cell interactions play critical roles. This could limit the translatability of our findings to more physiologically relevant conditions. Future studies should be aimed at validating these results in primary microglial cells and in vivo models, which would provide a more comprehensive understanding of WSC's immunomodulatory effects and its potential therapeutic applications in neuroinflammatory diseases.

While our findings offer valuable insights into the mechanistic underpinnings of WSC-induced trained immunity in microglia, they also underscore the necessity for further research. Future studies should be aimed at dissecting the intricate relationship between metabolic reprogramming and epigenetic changes in trained immunity more deeply. Additionally, examining the long-term functional outcomes of such trained immunity in microglia, particularly in the context of neuroinflammatory diseases, could elucidate potential therapeutic applications of WSC in modulating innate immune responses within the CNS.

In conclusion, our study presents the first evidence of WSC's ability to induce a trained immunity-like phenotype in microglial cells, characterized by significant shifts in metabolic and epigenetic profiles. The observed metabolic shift towards lactate production, coupled with epigenetic histone modifications, suggests a complex regulatory mechanism through which WSC primes microglia for enhanced inflammatory responses. These epigenetic alterations facilitate greater accessibility to the promoters of key inflammatory genes, such as cytokines and iNOS, signaling a new understanding of how trained immunity can be modulated in microglial cells.

## Data Availability

All the data produced and analyzed while performing this study are present in the article. However, any further inquiry can be made directly to the corresponding author.
